# Effects of spaceflight and simulated microgravity on microbial growth and secondary metabolism

**DOI:** 10.1186/s40779-018-0162-9

**Published:** 2018-05-14

**Authors:** Bing Huang, Dian-Geng Li, Ying Huang, Chang-Ting Liu

**Affiliations:** 1 Nanlou Respiratory Diseases Department, Chinese PLA General Hospital/Chinese PLA Postgraduate Medical School, Beijing, 100853 China; 2 State Key Laboratory of Microbial Resources, Institute of Microbiology, Chinese Academy of Sciences, Beijing, 100101 China

**Keywords:** Microbial growth, Secondary metabolism, Spaceflight, Microgravity, Simulated microgravity, Microgravity analogs

## Abstract

Spaceflight and ground-based microgravity analog experiments have suggested that microgravity can affect microbial growth and metabolism. Although the effects of microgravity and its analogs on microorganisms have been studied for more than 50 years, plausible conflicting and diverse results have frequently been reported in different experiments, especially regarding microbial growth and secondary metabolism. Until now, only the responses of a few typical microbes to microgravity have been investigated; systematic studies of the genetic and phenotypic responses of these microorganisms to microgravity in space are still insufficient due to technological and logistical hurdles. The use of different test strains and secondary metabolites in these studies appears to have caused diverse and conflicting results. Moreover, subtle changes in the extracellular microenvironments around microbial cells play a key role in the diverse responses of microbial growth and secondary metabolisms. Therefore, “indirect” effects represent a reasonable pathway to explain the occurrence of these phenomena in microorganisms. This review summarizes current knowledge on the changes in microbial growth and secondary metabolism in response to spaceflight and its analogs and discusses the diverse and conflicting results. In addition, recommendations are given for future studies on the effects of microgravity in space on microbial growth and secondary metabolism.

**Electronic supplementary material:**

The online version of this article (10.1186/s40779-018-0162-9) contains supplementary material, which is available to authorized users.

## Background

Microbes are highly evolved [1] and can survive in many extreme environments [2, 3], including outer space [4, 5]. However, the different mechanisms by which they respond and adapt to these environments (especially to microgravity in space) remain unclear. Recently, spaceflight and ground simulated microgravity (SMG) or low-shear modeled microgravity (LSMMG) experiments have demonstrated that microgravity can affect cellular processes and functions in microorganisms, such as cell growth [6–9], gene expression [10–12], cell morphology and development [13, 14], virulence and resistance [15–18], biofilm formation [19, 20], secondary metabolism [21–23], and microbial mutations and relation to adaptation to LSMMG [24]. Considerable effort has been focused on cell growth and secondary metabolism.

The significance of exploring the effects of space microgravity on microbial growth and metabolism includes two important implications. First, the growth of microorganisms (especially pathogenic microbes) in a space capsule could be a threat to astronaut health and be detrimental to their immune systems [10, 11, 25, 26]. Second, microorganisms can produce many special secondary metabolites that could be utilized as medicine for both humans and animals [5, 23, 27] as well as some toxic secondary metabolites that may threaten the health of astronauts [28]. Investigations into whether the production of secondary metabolites by these microorganisms is altered in the space environment are worthwhile.

Although studies on the responses of microbes to microgravity date back to the 1960s, many basic questions concerning the effects of microgravity on microbial behavior are far from being fully resolved [29, 30]. Moreover, our systematic and in-depth understanding of the genetic and phenotypic responses of a variety of microorganisms to microgravity environments in space is insufficient due to technological and logistical hurdles. To date, only a few typical microbes, including streptomycetes, have been investigated in terms of their responses to microgravity and its analogs [29]. Interestingly, plausible but conflicting results for cellular growth rates were reported in different spaceflight and clinorotation experiments [31]. In addition to the microbial growth rate, secondary metabolism was also found to be similarly sensitive to microgravity and simulated microgravity [22, 31]. Furthermore, the results of these studies have been mixed, without conclusive assertions and suggestions for future antibiotic production in space environments [22, 32]. Thus, nothing conclusive or concrete is known about the effects of microgravity or simulated microgravity on microbial growth and secondary metabolism; thus, this area of research remains open to further exploration.

In this review, we compare the technological methods of microgravity experiments used for spaceflight and ground-based simulated microgravity. We also analyzed the similarities and differences in their effects on microbial growth and secondary metabolism as well as the causes of the inconsistent results. Based on the analysis of previous studies, it is clear that the experiments performed under spaceflight and SMG conditions differed in some procedures, including in the use of different strains, growth media, and types of ground-based facilities (GBFs), which may lead to conflicting results. We also propose that subtle differences in the microenvironment could play a key role in the diverse responses observed for microbial growth and secondary metabolism. Finally, we provide recommendations for future studies on the effects of microgravity in a space environment on microbial growth and secondary metabolism.

## Space microgravity and its analogs on the ground

A large proportion of the experiments were performed under simulated microgravity conditions using ground-based microgravity simulators due to the scarcity and costliness of spaceflight opportunities. However, it should be noted that the real microgravity in space is not equivalent to microgravity analogs using ground-based simulators. Therefore, questions remain concerning the similarities and relationships between real space microgravity and simulated microgravity by ground-based simulators.

The use of the term “microgravity” in most studies refers to the conditions of “weightlessness” or “zero-g” that only exist in a space environment. In fact, microgravity is labeled “μg”’, referring to the fact that the gravitational forces are not entirely equal to zero but are just very small and that its corresponding “microgravity” level (i.e., the value of equivalent accelerated speed) needs to be specified in this context. Generally, the “microgravity” level ranges from approximately 10^− 3^ to 10^− 6^ *g* and is dependent on the location within the spacecraft and the frequency of vibrations [33, 34]. Therefore, the term “microgravity” has been suggested to be exclusively restricted to experiments performed in an environment such as drop towers, parabolic flights, sounding rockets, recoverable satellites, spaceships, and the space station (spacelab). Furthermore, the usage of the term “microgravity” should be independent of the interfering factors of the actual acceleration of the spacecraft in space and cosmic radiation, while the term “spaceflight” should contain “microgravity” and the other inherent factors in space (i.e., cosmic radiation).

Generally, each type of spaceflight opportunity has its own time range of duration and corresponding “microgravity” level based on the various spaceflight technologies [35] (Table 1). To date, many studies associated with the responses of terrestrial life have been conducted in space microgravity conditions by recoverable satellites, space shuttles and on the space station (spaceship) [4]. The effect of an organism in response to the microgravity of a space experiment in these studies is frequently described as the “spaceflight effect” due to considerations of the interference of cosmic radiation, spacecraft vibrations and hypervelocity; the effects of microgravity and spaceflight are different. Earlier studies often lacked on board controls during spaceflight due to restrictions in the use of centrifuges and sample fixation in orbit. Recently, these drawbacks have been gradually overcome by using an incubator-centrifuge in orbit that could simulate 1 *g* Earth gravity and thus separate other space environmental factors during spaceflight. Furthermore, real-time sample fixation in orbit could avoid the interference of spacecraft landing [36, 37].

**Table 1:** Several flight opportunities and their characteristics

Flight opportunities	Time of duration	Gravity level (*g*)
Drop tower	2–9 s	10^− 5^ - 10^− 2^
Parabolic flight	15–30 s	10^− 3^ - 10^− 2^
Sounding rockets	6–15 min	10^− 4^ - 10^− 3^
Recoverable satellites/space shuttle	1–2 mon	10^− 5^ - 10^− 3^
Space station (spacelab)	Several years or permanent	10^−6^ - 10^− 5^

Although some of these studies were conducted in a space environment by means of the spacecraft and space station, microgravity experiments in space are costly and performed infrequently due to technological and logistical hurdles. Hence, several GBFs with different physical concepts have been constructed to simulate microgravity on the ground [38, 39] (Table 2). The term “simulated microgravity” emerged from microgravity analogs using GBFs. It has been suggested that the term should be used only for experiments performed in GBFs in which the direction of the gravity vector has undergone a continuous and constant change, with the gravity level averaged to near zero along with rotation and time [38]. Indeed, real microgravity cannot be achieved with a ground-based simulator because the magnitude of the Earth’s gravity vector cannot be changed, although its effect can be changed. In this respect, the microgravity analog of “simulated microgravity” created by GBFs has been regarded as “functionally near weightlessness” and was not equal to the “μg” in space or “weightlessness”. In other words, the effects generated by GBFs may be similar to those of microgravity in space, and preliminary experiments before launch should first be performed using GBFs. Here, several GBFs that are frequently used are described.

**Table 2:** Several ground-based facilities (GBFs) and their characteristics

Ground-based facilities (GBFs)	Simulative effect	Suitable organism
2-D clinostats	Simulated microgravity effect	Plant tissue
Random positioning machines (RPMs) (3-D clinostats)	Simulated microgravity effect	Plant tissue
High-aspect rotating vessels (HARVs)	Low-shear modeled microgravity (LSSMG)	Human cells, animal cells, microorganisms
Rotating-wall vessel (RWV)		
Rotating-wall bioreactor (RWB)		
Rotary cell culture system (RCCS)		
Diamagnetic levitation apparatus	Simulated microgravity	Protozoan, plants, mammals, microorganisms

### Clinostats

In its early stages, the clinostat was a very well-established paradigm to simulate microgravity on the ground [40]. In principle, a clinostat is a device that enables the rotation of the samples with one or two axes and prevents the organism from perceiving the gravity vector by continuously breaking the direction of the gravity vector. The number of rotational axes, the speed, and the direction of rotation were designed according to different organisms and experimental requirements for the practical application of clinostats [41–43]. Two-dimensional (2-D) clinostats rotate perpendicularly to the direction of the gravity vector, with one rotation axis representing a classical and well-established model to simulate microgravity; these clinostats are widely applied to study the effects of microgravity on biological samples today. Moreover, several studies have shown that the results obtained from various model systems using 2-D clinorotation were similar to those found under real microgravity conditions [44–46].

### Random positioning machines (RPMs)

A clinostat with two axes is called a three-dimensional (3-D) clinostat. The use of 3-D clinostats was hypothesized to be capable of increasing the quality of simulations, especially for larger organisms. Often, 3-D clinostats rotate biological samples along two independent axes to change their orientations at constant speeds and directions relative to the gravity vector, thereby eliminating the effect of gravity. Specifically, 3-D clinostats that rotate with random changing speed and direction relative to the gravity vector are called “RPMs” [40, 47]. There is increasing evidence that the use of the RPMs can generate effects similar and comparable to the effects of real microgravity in space when they rotate fast enough that the organism cannot perceive and experience the gravity vector (i.e., the changes in direction are faster than the organism’s minimum response time (MRT) to the gravity vector). Relatively responsive living organisms, such as plants and other higher organisms, have been observed to be more suitable and ideal candidates for investigations involving RPMs [48].

### Rotating wall vessels (RWVs)

The RWVs were initially developed by the NASA Johnson Space Center (Houston, TX, USA) for cell cultures. RWVs can generate LSMMG and have been used as an optimized suspension culture technology [49, 50]. Usually, RWVs consist of a hollow disk or cylinder that is entirely filled with a liquid medium with almost no bubbles; this disk or cylinder rotates perpendicularly to the direction of the gravity vector with one rotational axis. The cells are maintained in suspension under special culture conditions when the RWVs run in solid-body rotation, establishing a continuous low-shear, low-turbulence environment for cell growth that is similar to the space microgravity environment. RWV analogues, such as rotating wall bioreactors (RWBs), rotating cell culture systems (RCCSs) and high-aspect rotating vessels (HARVs), were designed according to similar physical principles using different configurations and have also been frequently applied to study the effects of microgravity on biological samples [39].

### Diamagnetic levitation

Diamagnetic levitation is an emerging technology that uses a strong, spatially varying magnetic field produced by a Bitter solenoid or a superconducting solenoid magnet to simulate an altered gravity environment and generate aspects of weightlessness similar to the conditions observed in space [38]. Interestingly, the diamagnetic force opposes the force of gravity on a levitating object, similar to the manner in which the centrifugal force balances the gravitational force on an orbiting spacecraft. The purpose of diamagnetic levitation is to lessen the internal stresses induced by the force of gravity to as close to zero as possible to simulate a near weightless environment. Normally, the diamagnetic repulsion of the object or living organism exactly balances its gravity throughout the body. This was demonstrated to make investigating the effects of weightlessness on small organisms feasible without going into space. In previous studies, diamagnetic levitation was used to successfully levitate protozoans [51], plants [52], animals [53, 54], and microorganisms [55, 56]. Thus, the use of diamagnetic levitation as a ground-based tool has been frequently applied to investigate the effects of microgravity on living organisms, including cell growth and gene expression [55], secondary metabolism [57], and plasmid transfer [58]. Although some advantages have been demonstrated for diamagnetic levitation, it must be noted that the strong magnetic field itself may influence the organism and that the effects of simulated weightlessness may receive some interference from the strong magnetic field [57]. Accordingly, the effects of interference should be evaluated prior to the simulated microgravity experiment. Moreover, distinguishing between the effects of magnetically simulated weightlessness and any other effects of the strong magnetic field by performing careful control experiments is important.

Although the physical principles among these GBFs are not exactly the same, they can achieve a similar function of simulated microgravity on the ground. As discussed above, it should be noted that the physical principle of microgravity simulators on the ground is different from that of the real microgravity experienced in space, although the effects of microgravity on the organism can be simulated by microgravity analogs on the ground to a certain extent. Indeed, reports have indicated that the effects of “simulated microgravity” using GBFs on the organism are not all the same as the effects induced by microgravity in space [31]. However, an increasing number of studies have shown that the results from various model systems using 2-D clinostats, RPMs, RWV or RWB analogs, and diamagnetic levitation were similar to those found in real microgravity environments [38]. These results suggest that GBFs are feasible and cheap tools that can be used on the ground and that could play an important role in microgravity experiments. Moreover, the effects of “simulated microgravity” on the organism could be used as preliminary and screening experiments of the microgravity effects during spaceflight, and the “simulated microgravity” experiments performed using GBFs could also be used as the ground controls of the spaceflight or microgravity experiments in space.

## Microbial growth responses to spaceflight and simulated microgravity

An increasing number of studies have investigated the growth responses of bacteria, fungi and archaea to microgravity in space and to microgravity analogs on the ground. In these studies, the bacteria mainly included *Escherichia coli* [17, 24, 56, 59–71], *Bacillus subtilis* [7, 59, 67, 68], *Salmonella typhimurium* [18, 72], *Pseudomonas aeruginosa* [8, 73, 74], *Staphylococcus* [28, 56], *Streptococcus* [75], *Streptomyces* [76, 77], etc. [78–80]; the fungi mainly included *Saccharomyces cerevisiae* [13, 55, 81] and *Candida albicans* [25]; and the archaea included *Haloarchaea* [82]. An increasing amount of evidence has suggested that microorganisms are just as ubiquitous in space habitats as they are on Earth [83–93]. However, inconsistent results concerning cellular growth rates have frequently been reported in these studies. From a comprehensive analysis of these results in their contexts, we found that microbial growth responses to microgravity and its analogs were dependent on two dominating aspects. First, the selection of strains for these studies varied based on different experimental objectives, with the most inherent characteristic of these strains embodied in the property of cell motility. Second, the experimental conditions varied between studies, including in the microgravity conditions, culture methods (suspension and agar cultures), and the medium nutrient concentrations (high and low nutrient concentrations) (Additional file 1: Table S1).

### The growth effects are associated with the microbial species and strains used

As described previously, many investigations reported seemingly diverse results and effects on microbes exposed to microgravity and simulated microgravity [31]. In these investigations, the enumeration of the final cell population as an indicator of the growth rate was reported more frequently than any other measured variable for comparisons of the differences in the spaceflight cultures and controls. Generally, most studies found that spaceflight increased the microbial growth rate under microgravity and simulated microgravity conditions [7, 24, 59, 60]. However, several microbes exposed to simulated microgravity were reported to grow more slowly compared to the controls [76, 77], while other studies reported that no significant differences were found in the growth rates of microbes subjected to spaceflight cultures and those of the ground control group [67, 68]. The discrepant effects were largely dependent on the microbes and strains used in these studies. For example, *E. coli* ZK650 cultures were found to have a higher dry cell weight under LSSMG in the HARVs [65], while no differences were found in the dry cell weight of *Bacillus brevis* Nagano cultures in the HARV [94]. In addition, the streptomycetes (*Streptomyces clavuligerus* ATCC 27064 and *Streptomyces hygroscopicus* ATCC 29253) were both found to have lower dry cell weights in their cultures in the HARVs and RWBs, respectively [76, 77].

Interestingly, differences in growth effects under microgravity and its analog conditions have been reported for different strains of *E. coli* [56, 59, 62–71]. Surprisingly, different growth responses were demonstrated in some studies for the same strain of *E. coli* (*Escherichia coli* ATCC 4157) [7, 59, 67, 68]. The type strain of *E. coli* has been the frequent focus of investigations that showed that the microbial growth rate was increased under microgravity or its analog conditions. A series of experiments was performed using suspension cultures of *E. coli* aboard several US Space Shuttle missions. The results of these studies showed not only that the final cell population density of *E. coli* was approximately doubled in the spaceflight cultures but also that the lag phase was shortened and that the duration of exponential growth was extended [7, 59]. Other experiments performed under simulated microgravity conditions on the ground using RWVs, RWBs, HARVs, RCCSs and diamagnetic levitation indicated that the growth rate of *E. coli* was similarly increased [56, 62, 65, 66, 71, 78]. However, the *E. coli* growth rate was also found to be unchanged under microgravity or simulated microgravity conditions similar to those described in these studies [17, 63, 66, 71, 78]. In fact, a subsequent set of studies using *B. subtilis* found similar results in the exposure of cultures to microgravity during spaceflight [7, 59, 67, 68].

### The growth effects are associated with the strain’s inherent properties (cell motility)

The study of Baker et al. [78] provided substantial evidence that microbial growth under simulated microgravity conditions using RCCSs apparatus varied with the cellular motility of the strains used. In their study, simulated microgravity did not affect the motile strain *Sphingobacterium thalpophilium* (which had flagella) regardless of the method of enumeration and the medium used, while significantly higher numbers of the non-motile strain *Ralstonia pickettii* (which lacked flagella) were found in a high nutrient broth under simulated microgravity conditions compared to normal gravity conditions. Benoit et al. [31] reviewed the experimental results from previous investigations and suggested a strong correlation between the growth effects of bacteria grown in suspension cultures exposed to spaceflight or microgravity analogs on the ground and cell motility. This review indicated that spaceflight and microgravity analogs increased microbial growth of non-motile bacteria in suspension cultures. In light of these findings, recent studies regarding microbial growth under microgravity conditions and its analogs gradually took cell motility into consideration. However, an exception was recently reported, which examined the growth of *Pseudomonas aeruginosa* PA14 and its *ΔmotABCD* mutant, which is deficient in swimming motility [8]. As described in this study, the final cell densities observed with the motility mutant were consistent with those observed with the wild type during spaceflight.

### The growth effects are associated with the culture methods

In most studies, microbial growth experiments under microgravity or its analog conditions were performed using suspension cultures, while only a few studies were performed on solid or semi-solid media. Of the studies described above, microbial growth in suspension cultures was frequently shown to exhibit an increased final cell density under microgravity or its analog conditions, while no distinct differences in the final cell populations were found for growth on solid or semi-solid media [7, 59, 67, 68]. Previous spaceflight experiments demonstrated that suspension cultures of *E. coli* and *B. subtilis* exhibited increased cell growth in the spaceflight environment [7, 59, 69]. However, other studies showed that *E. coli* and *B. subtilis* grown on solid agar during Space Shuttle Mission STS-63 did not experience an increased final cell mass, but that changes in other growth characteristics might have occurred when the bacteria were grown under various gravitational conditions [67, 68]. In suspension cultures, cells are likely to experience microgravity both simulated and real, because they are on a “free fall” inside the suspension and subject to a gravity vector. However, in agar (solid) substratum, the cells are already “attached” to a surface, and the gravity vector effect cannot occur. This finding also indicated that fluid dynamics and extracellular transport phenomena but not cellular dynamics were the most likely causes of the previously reported increases in bacterial growth under microgravity conditions. Surprisingly, Van Mulders et al. [13] reported that the model eukaryotic organism *Saccharomyces cerevisiae* S1278b (laboratory strain) exhibited reduced invasive growth on semi-solid agar medium under microgravity conditions, while no differences in invasive growth were observed for the CMBSESA1 industrial strain.

**Fig. 1:**
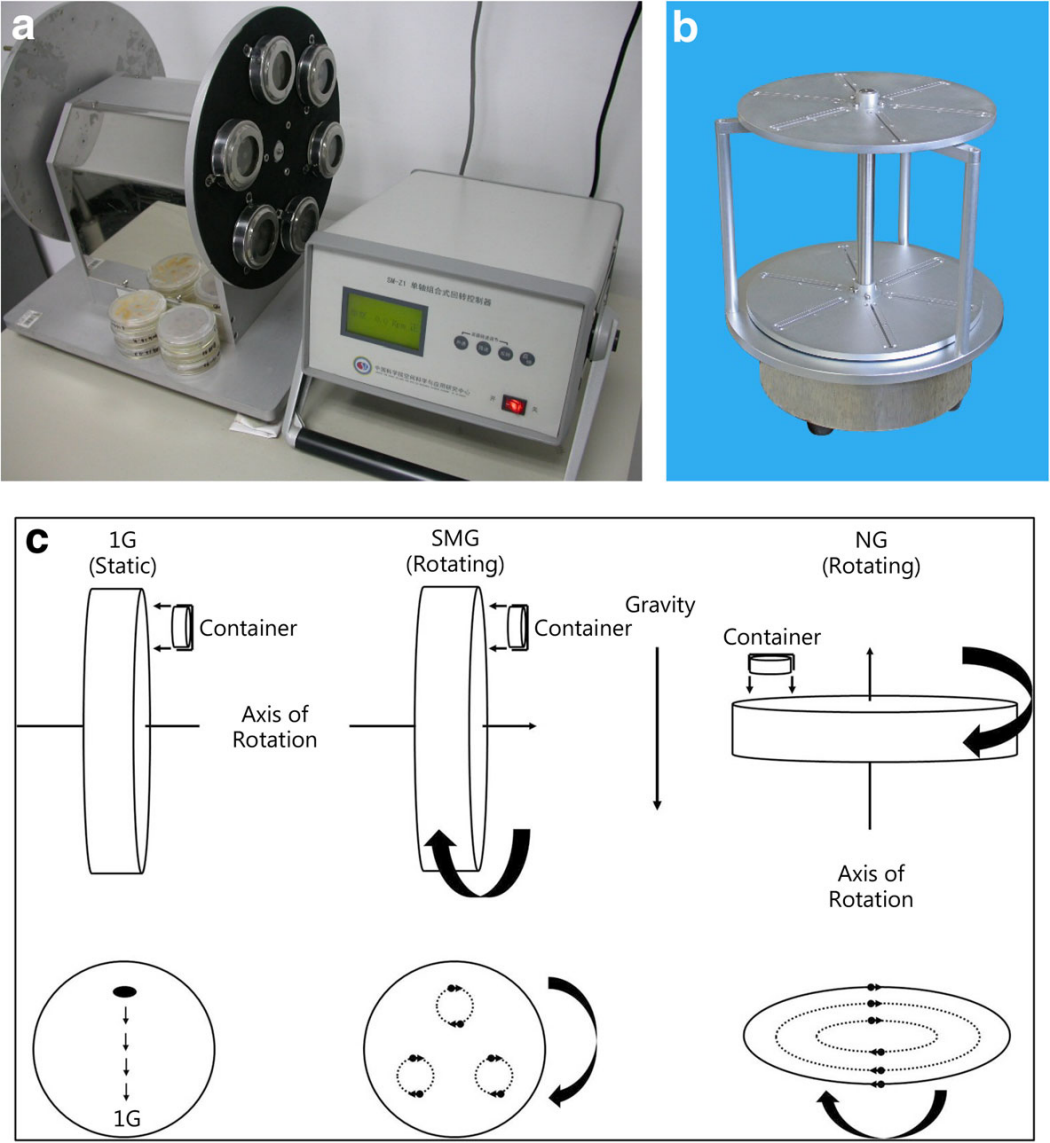
The clinostats used in the SMG experiments and their corresponding principle models. **a** The 2-D clinostats with a horizontal axis used to generate SMG condition on ground. **b** The 2-D clinostats with a vertical axis used to generate 1G control condition on ground. **c** The schematic diagram of mechanical principle of the different clinostats. SMG: Axis of rotation is perpendicular to the direction of the gravity vector, which is used to simulate microgravity effects on ground; NG: The samples fixed in the metal support are rotating, used as the dynamic control of SMG; 1G: The samples fixed in the metal support are static and are used as the static control of SMG

**Fig. 2:**
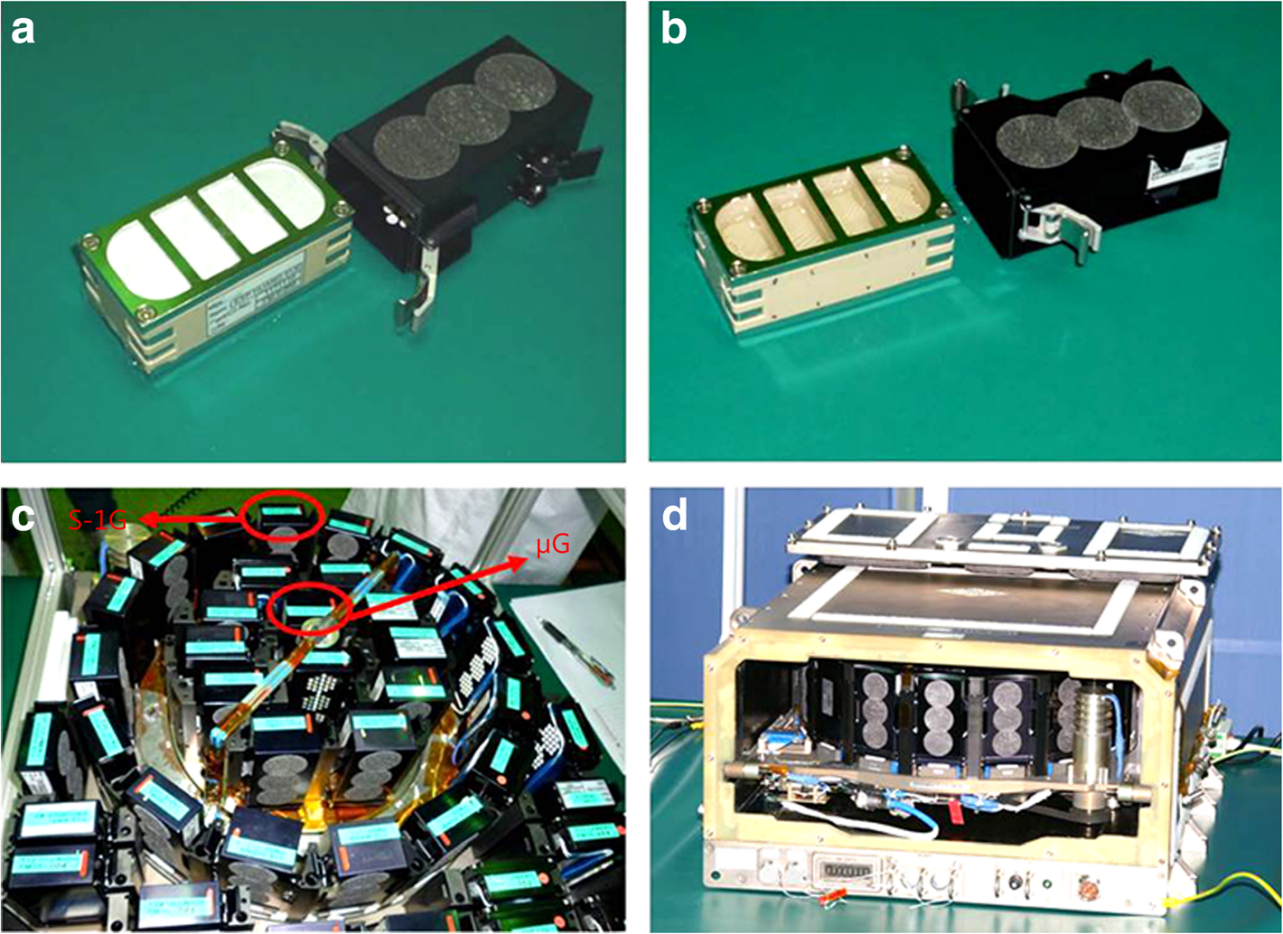
Shenzhou-8 spaceflight experiments. Microbial samples cultured in solid (**a**) and liquid (**b**) media were loaded into Experiment Unique Equipments (EUEs) before launching. **c** The inside of SIMBOX. Red circles indicate the two EUEs in a static slot (microgravity position, μG) and a centrifuge slot (simulated 1G position, S-1G), respectively. **d** SIMBOX as an advanced space incubator

**Fig. 3:**
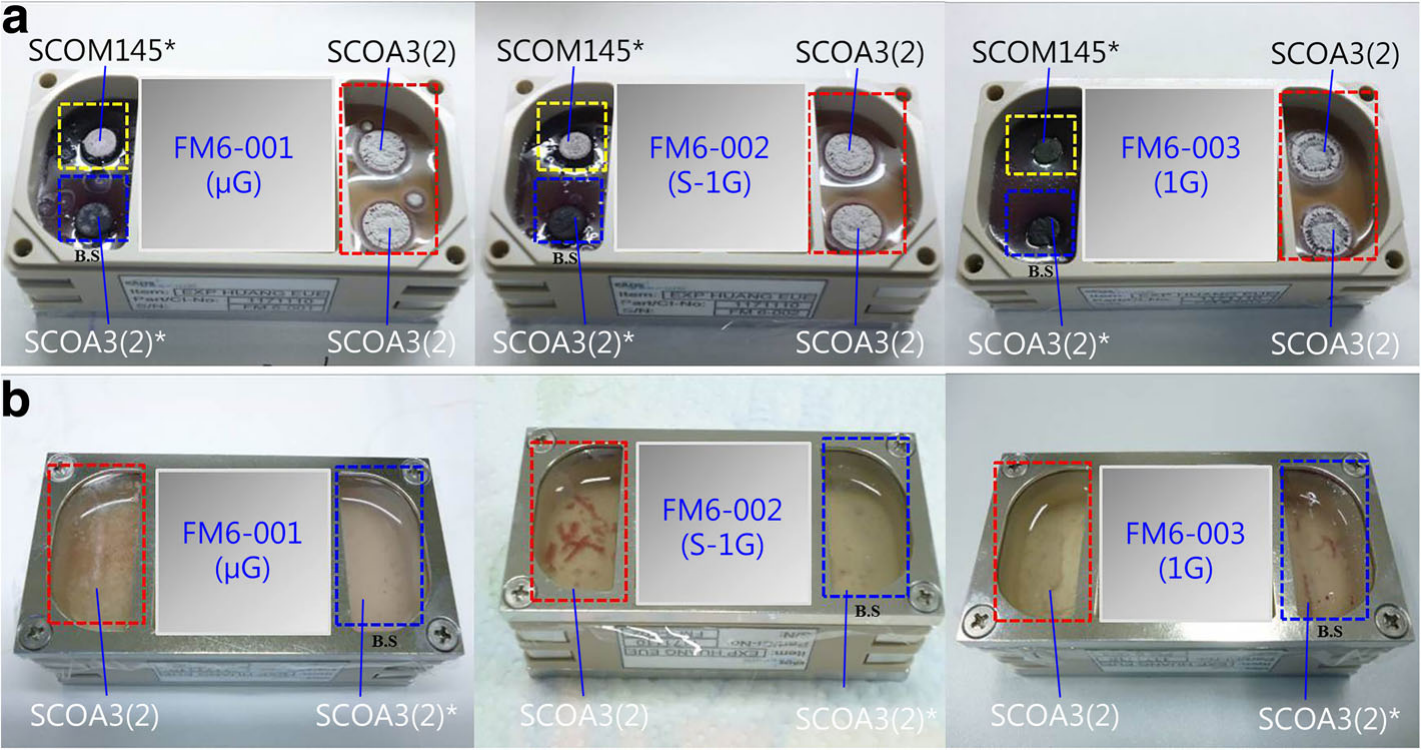
Colony and cultural features of *S. coelicolor* after the 16.5-day spaceflight experiment [95]. **a** Colony features after the 16.5-day spaceflight experiment. “SCOA3(2)” framed in red indicates the pure *S. coelicolor* A3(2) culture, “SCOA3(2)*” framed in blue indicates the *S. coelicolor* A3(2) co-cultured with the indicator strain *B. subtilis*, and “SCOM145*” framed in yellow indicates *S. coelicolor* M145 co-cultured with *B. subtilis*. **b** Cultural features of *S. coelicolor* A3(2) grown in a JCM42 liquid medium after the 16.5-day spaceflight experiment. “SCOA3(2)” framed in red indicates the pure *S. coelicolor* A3(2) culture, and “SCOA3(2)*” framed in blue indicates *S. coelicolor* A3(2) co-cultured with *B. subtilis*

Our lab investigated the effects of spaceflight and SMG (Fig. 1) on the growth of *Streptomyces coelicolor* A3(2), which were incubated in “SIMBOX” (Science in Microgravity Box, Astrium, Germany) during the Shenzhou-8 space mission (μG = 10^− 3^–10^− 4^ *g*). SIMBOX (Fig. 2) is an advanced space incubator with 42 separate EUEs (Experiment Unique Equipments, Astrium, Germany) for experimental containers and a 1 *g* centrifuge to simulate gravity in space [36, 37]. Our results showed that the growth rate of strains cultured on yeast-starch agar medium (JCM42) was not influenced by either SMG or spaceflight; however, the cell biomass grown in liquid cultures was increased under SMG or spaceflight conditions (Fig. 3) [95]. The increased final cell biomass under both SMG and spaceflight conditions was found to be due to the fluidic turbulence of the liquid medium, which did not fill the whole culture chamber. These results are similar to those obtained from previous studies on *E. coli* and *B. subtilis*, which also showed that the latter two microorganisms grew faster and yielded more biomass in liquid suspension cultures under microgravity conditions during spaceflight [7, 69] but exhibited no visible differences in growth rates in agar or semi-solid cultures [67, 68]. The growth rate of the strains was speculated to be related to fluid dynamics and the distribution of the liquid medium rather than to cellular effects induced by the microgravity environment. Despite the fact that it is difficult to interpret the puzzling results described in these studies, the culture conditions of microorganisms in microgravity experiments should be emphasized due to their importance for the effects of microgravity.

### The growth effects are associated with the nutrition concentrations and oxygen availability of the liquid media

Interestingly, recent studies have demonstrated that the effects of microgravity or its analogs on microbial growth differed when the microbes were cultured in liquid media with high or low nutrient levels. Baker et al. [6] found that there were few differences in the cell numbers and that the cell size was not affected by the modeled reduced gravity when *E. coli* was grown in minimal medium. However, the total cell numbers were higher and cells were smaller when grown under reduced gravity in higher nutrient conditions compared to those of the normal gravity controls. In summary, this study revealed that the growth responses to modeled reduced gravity varied with nutrient conditions; the authors also speculated that larger surface-to-volume ratios may have helped to compensate for the zone of nutrient depletion around the cells under modeled reduced gravity.

In the study of Kim et al. [8], wild-type *Pseudomonas aeruginosa* PA14 grown during spaceflight showed an increased final cell density compared to the normal gravity controls when low concentrations of phosphate in the medium were combined with decreased oxygen availability; in contrast, no difference in the final cell density was observed between spaceflight and normal gravity when the availability of either phosphate or oxygen was increased. These results indicated that differences in the final bacterial cell density observed between spaceflight and normal gravity cases were due to an interplay between microgravity conditions and the availability of substrates essential for growth. Similarly, a study by Dijkstra et al. [56] showed that diamagnetic levitation increased the growth rate and reduced the sedimentation rate in liquid cultures of both *Escherichia coli* K12 MG1655 and *Staphylococcus epidermidis* NCTC11047. Further experiments revealed that the increased growth rate was due to enhanced oxygen availability as a result of convective stirring in the liquid induced by the magnetic field.

### The growth effects are associated with the rotation speed of the simulator

Recent studies demonstrated that the effects of microgravity or its analogs on microbial growth were different when the microbes were cultured at higher speeds and lower speeds in the RCCS. A study by Baker et al. [6] showed that significant differences in the final cell populations between modeled reduced gravity in the RCCSs and normal gravity controls were observed only at higher speeds (30–50 r/min) when *E. coli* ATCC 26 was subjected to different rotation speeds during clinorotation. However, the differences were found to be non-significant in the final cell populations grown at lower speeds (less than 20 r/min).

Factors other than microgravity may influence microbial growth in space. Cosmic radiation and slightly elevated CO_2_ levels are both other factors influenced by spaceflight, and the effect of spaceflight is an integration of multiple results in response to space-based factors. Generally, it is difficult to distinguish the factor from which the effect comes. The biological effects of cosmic radiation may be induced through direct energy absorption. For example, DNA is damaged after exposure to ionizing radiation, leading to an increase in mutation. However, these effects are also induced indirectly via interactions of those molecules with radiation-induced radicals, also leading to an increase in cell damage. During spaceflight, microgravity could interfere with the operation of the cellular repair processes of DNA when damaged by cosmic radiation, bringing an increase in the radiation response [4]. Some microbial cells that are susceptible to slightly elevated CO_2_ levels in spacecraft could change their growing status; thus, the effect induced by the elevated CO_2_ levels is likely to dominate all responses. Therefore, other factors, such as cosmic radiation and slightly elevated CO_2_, may also lead to conflicting results in microbial growth.

Taken together, the different effects observed using the same species in these studies may be due to differences in the species’ inherent attributes and the experimental conditions. The strain’s inherent attributes were mainly specified according to cell motility [31, 78]. Additionally, the extrinsic experimental conditions were speculated to play a role in microbial responses. In other words, it is highly likely that the cellular surroundings affect their growth responses to microgravity and its analogs on the ground [59, 67, 68]. It was hypothesized that indirect physical effects, such as changes in fluid dynamics and extracellular transport, rather than a direct microgravity effect were the most likely cause of the differences observed in bacterial growth during spaceflight. Thus, the extracellular surroundings represented by the microenvironments encountered by the organisms varied under different experimental conditions in previous studies (i.e., the culture mode, the medium nutrient concentration, and the rotation speed). The majority of the proposed mechanisms focused on physical factors, such as decreased mass diffusion or shear levels or the development of microenvironments (i.e., changes in the distribution of nutrients and by-products due to a lack of cell sedimentation) that directly affected the organisms and therefore could alter cell growth.

Although conflicting results of the growth effects following the exposure of microbes to spaceflight or its analogs on the ground were found for the different strains used in these studies, the microenvironments around the cells should be considered in the interpretation of the results and phenomena. In our opinion, external physical factors would play a more dominant role in the effects of microbial growth under spaceflight or its analog conditions.

## Effect of spaceflight and simulated microgravity on microbial secondary metabolism

Generally, the biosynthesis and yield of microbial secondary metabolites are sensitive to extracellular environmental signals and stresses, including nutrients, heat, osmotic stress and shear stress [96–98]. Therefore, it would be interesting to look into the yield of secondary metabolites produced in the cultures under microgravity and its analog conditions [5]. Similar to the effects of the conditions on the microbial growth rate, diverse results involved with the yields of secondary metabolites were frequently reported [22, 32], which are summarized in Table 3. A review of these responses is both significant and timely for scientists considering the application of simulated microgravity on the ground to explore the effects of the space microgravity environment on secondary metabolites, many of which are used in human and veterinary medicine (i.e., antibiotics, anti-tumor agents and immunosuppressants) [99].

**Table 3:** Responses of the yields of secondary metabolites to simulated microgravity and spaceflight

Strain	Antibiotics	Response	Equipment
*Streptomyces clavuligerus* ATCC 27064 [77]	β-lactam antibiotic cephalosporin	Inhibited	High-aspect rotating vessels (HARV)
*Streptomyces hygroscopicus* ATCC 29253 [76]	Rapamycin	Inhibited	Rotating-wall bioreactor (RWB)
*Streptomyces ansochromogenus* 7100 [100]	Nikkomycin X, Z	Increased	Unmanned satellite
*Streptomyces avermitilis* PE1 [57]	Avermectin	Increased	Diamagnetic levitation
*Streptomyces plicatus* WC56452 [27]	Actinomycin D	Increased	US Space Shuttle mission STS-80
*Streptomyces plicatus* WC56452 [5]	Actinomycin D	Increased and then decreased	International space station (ISS)
*Escherichia coli* ZK650 [65]	Microcin B17	Inhibited	High-aspect rotating vessels (HARV)
*Bacillus brevis* Nagano [94]	Gramicidin S	Unaffected	High-aspect rotating vessels (HARV)
*Humicola fuscoatra* WC5157 [23]	Monorden	Increased	US Space Shuttle mission STS-77
*Microcystis aeruginosa* PCC7806 [101]	Microcystin	Increased	Rotary cell culture system (RCCS)
*Cupriavidus metallidurans* LMG 1195 [21]	Poly-β-hydroxybutyrate (PHB)	Increased and then decreased	Rotating wall vessel (RWV)
*Streptomyces coelicolor* A3(2) [95]	Undecylprodigiosin (RED)	Increased slightly	2D-clinostat (SM-1)
*Streptomyces coelicolor* A3(2) [95]	Actinorhodin (ACT)	Inhibited	2D-clinostat (SM-1), Shenzhou-8 Space mission

### The yields of secondary metabolites are in/de-creased or uninfluenced in response to spaceflight and simulated microgravity

In the study of Lam et al. [27], the specific productivity (pg/CFU) of actinomycin D produced by *Streptomyces plicatus* WC56452 was increased during spaceflight aboard the US Space Shuttle mission STS-80. In another study [23], Lam reported that the production of monorden by *Humicola fuscoatra* WC5157 grown on two types of agar media (T8 and PG) was also increased during spaceflight aboard the US Space Shuttle Mission STS-77. In a study by Luo et al. [100], the productivity of nikkomycins by *Streptomyces ansochromogenus* increased by 13–18% during 15 days of spaceflight aboard a satellite, and the proportion of nikkomycin X and Z increased correspondingly. A study by Xiao et al. [101] demonstrated that the production of the toxin microcystin (MC) by the cyanobacteria *Microcystis aeruginosa* PCC7806 was enhanced by simulated microgravity (SMG), which acted as a novel environmental signal, while its growth was inhibited. Moreover, enhanced MC production was reported to be associated with pigment and nitrogen metabolism. Liu et al. [57] reported that the production of the important anthelmintic agent avermectin produced by *Streptomyces avermitilis* in a solid culture was increased in an altered gravity environment simulated by diamagnetic levitation and under a strong magnetic field. However, this study indicated that the magnetic field was a more dominant factor in influencing changes in secondary metabolite production than altered gravity.

In the studies of Fang et al. [76, 77], the production of the β-lactam antibiotic cephalosporin and the polyketide macrolide rapamycin by *S. clavuligerus* ATCC 27064 and *S. hygroscopicus* ATCC 29253, respectively, were shown to be inhibited by LSMMG. Further analysis showed that growth under LSMMG conditions favored the extracellular production of rapamycin. Moreover, the site of rapamycin accumulation was modified to a moderate extent towards an extracellular location, while the total yields of rapamycin were decreased. Fang et al. [65] also demonstrated that the production of the peptide antibiotic microcin B17 by *E. coli* ZK650 was inhibited by LSMMG in HARVs. The site of microcin accumulation was found to be markedly different depending on whether *E. coli* was grown in shaking flasks or RWBs. The accumulation of microcin was intracellular when the bacteria were grown in flasks, whereas in HARVs, the majority of the microcin was found in the extracellular medium. It should be noted that the shift in the localization of microcin from intracellular to extracellular was probably due to the much lower degree of shear stress in the bioreactors because the addition of a single glass bead to the RWB medium created enough shear to change the site of microcin accumulation from the medium to the cells [65].

In another study by Fang et al. [94], gramicidin S (GS) production by *B. brevis* Nagano in NASA HARVs was found to be unaffected. Interestingly, this finding indicates that LSMMG does not have a universally negative effect on secondary metabolism and suggests that microbes respond to LSMMG in specific ways.

### The yields of secondary metabolites fluctuate over time in response to spaceflight and simulated microgravity

In the study of De Gelder et al. [21], the yield of poly-β-hydroxybutyrate (PHB) produced by *Cupriavidus metallidurans* LMG 1195 under LSSMG conditions in the RWVs was reported to be increased after 24 h of culture, while after 48 h of culture, the PHB concentrations were reduced in SMG compared to in the control. In the study of Benoit et al. [5], the production of actinomycin D by *Streptomyces plicatus* WC56452 in space was reported to be increased by 15.6 and 28.5% on days 8 and 12, respectively, but decreased in all subsequent matched sample points (16–72 d at intervals of 4 d) compared to the ground controls. Finally, the maximum production levels were found at day 24 in the ground control and at day 12 in the spaceflight samples, respectively.

Collectively, these studies suggest that microgravity or its analogs on the ground could alter secondary metabolisms in microorganisms. However, corresponding molecular evidence for the metabolic phenotypes has not been reported. Our latest study revealed the effect of simulated microgravity and spaceflight on the secondary metabolism of *S. coelicolor* A3(2) at both the phenotypic and whole transcriptome levels [95]. For *S. coelicolor* A3(2), the secondary metabolite undecylprodigiosin (RED) was produced earlier and accumulated to a slightly higher concentration under SMG conditions in agar culture compared to the ground control, while the production of actinorhodin (ACT) was delayed and markedly decreased. The gray spore pigment TW95a (*whiE*) accumulated faster and higher under SMG conditions than under 1 *g*. During spaceflight, the production of RED and ACT both decreased, while TW95a accumulated to a concentration higher than the control. The phenotypic responses of secondary metabolites were further supported by the whole transcriptome and qRT-PCR analysis [95].

Based on both previous studies and our studies and considering the complex regulatory network involved in antibiotic production, it is highly likely that the alteration of the secondary metabolism of streptomycetes by microgravity is strain-, medium-, pathway- and/or case-specific and therefore lacks directed and consistent behavior. As discussed above, the extracellular microenvironment around microbial cells is involved in the processes of their growth responses to microgravity and its analogs on the ground and is likely also involved in their secondary metabolism responses. Similarly, it should be noted that the responses of microbial secondary metabolism to microgravity are likely to be due to the indirect physical effects of microgravity, such as changes in fluid dynamics and the extracellular transport of metabolites. For suspension cultures, fluid dynamics may be more responsible for the effects of simulated microgravity on microbial cells, while for solid cultures, the gas dynamics in the cultivating vessel may influence the effects of simulated microgravity on microbial cells. Hence, based on the abovementioned studies and theory, it is reasonable to envision that the responses of microbial secondary metabolism to microgravity could be interpreted and elucidated in this way. Various extracellular stress signals have been found to induce or promote secondary metabolisms in a variety of microbes, and the extracellular stresses are transferred to the downstream responsive genes by a cascade of complex signal transduction steps [96–98]. Thus, despite the fact that the extracellular stress signals induced by microgravity were the same, the processes and steps experienced were different for different secondary metabolites when the extracellular stress signals were transmitted to the pathway-specific regulatory gene of the specific secondary metabolite. Meanwhile, the extracellular microenvironment affects the secretion and transport of secondary metabolites. Furthermore, the cellular surroundings (i.e., microenvironment) in different experiments may have contributed to these differences. For the experiments under ground-based microgravity analog conditions, the use of various types of GBFs with different physical concepts contributed to the observed differences in effects, most likely as a result of the different extracellular microenvironments around the microbial cells. In summary, the differences in the effects on microbial secondary metabolism were largely due to both the different secondary metabolites assayed in the test strains and the subtle differences in the microenvironments around the microbial cells; these factors collectively influenced the final results.

Although it is true that these studies have provided substantial evidence that microorganisms alter their secondary metabolic properties under spaceflight and ground-based analog conditions, the specific cause-and-effect mechanisms of the microbial secondary metabolism responses to microgravity remain unclear. To identify specific cause-and-effect pathways involved in the effects of microgravity on microbial secondary metabolism, the analysis of the corresponding gene expression and regulation will be indispensable, and the microenvironments around the microbial cells should be emphasized and characterized in future studies. This approach will help to develop an in-depth understanding of the life processes of microorganisms under space microgravity as well as earth gravity.

## Conclusions

With the human exploration of space accelerating, the growth and metabolic responses of microorganisms to the extreme environment of space are becoming the subjects of increasing concern. The progress of spaceflight and ground-based simulated microgravity technology has promoted our understanding of the effects of microgravity in space on microbes. It should be noted that there are several cases in which the effects of simulated microgravity using GBFs are not the same as the effects of exposure to microgravity during spaceflights. Additionally, there may be major influencing factors other than microgravity that can affect the growth and metabolisms of microorganisms during spaceflight, such as cosmic radiation and the vibrations generated by the rocket and acceleration during the launch and landing of the spacecraft [4]. Meanwhile, cosmic radiation is an important interfering factor that results in a difference in the effects of spaceflight and SMG using GBFs because there is often no cosmic radiation condition in the latter case.

Currently, it is widely believed that the responses of cells to gravity are attained by three possible pathways. The first is based on the actions of a special molecules or organelles that function as a gravireceptor (i.e., the “direct” effect). The second is based on the adaptive response of the cells to the changing microenvironment (i.e., the “indirect” effect), including extracellular nutrient distribution and transport of metabolites by fluid dynamics. The third is based on the actions of the integrated effects of the first two pathways--the “bifurcation theory” (symmetry breaking) [102, 103]. It should be noted that this theory was largely based on studies of higher organisms (i.e., plants and animals). Microorganisms are low and simple lifeforms that do not possess a gravireceptor, unlike higher plants and animals [104, 105]; however, they could respond to changes of gravity. Thus, the microorganisms may respond to microgravity or reduced gravity via the changing microenvironment in the medium. An increasing number of studies have shown that spaceflight and simulated microgravity experiments induced alterations in the growth rates and the productions of secondary metabolites as well as global alterations in gene expression, protein regulation, and transport of metabolites. The complicated alterations were correlated with microbial adaptation to microgravity conditions; in addition to the specific cell motility and secondary metabolites and pathways, changes in the extracellular microenvironment around the microbial cells induced different responses.

To predict the growth behavior and response of pathogenic bacteria and the production of microbial drugs such as antibiotics on long-term missions in space, the effect of microgravity and its analogs on microbial growth and secondary metabolism should be intensively studied, and the specific cause-and-effect mechanisms of microbial responses to microgravity should be disclosed at the molecular level. For preciseness, future studies should take two important aspects into consideration: the strains used and their cell motility properties as well as the microenvironment around the microbial cells within the given experimental conditions, such as the culture methods (suspension culture or agar culture), medium nutrient concentrations (high or low nutrient concentration), and rotation speeds (fast rotating or slow rotating).

## Additional file

Additional file 1: The different responses of microbial growth to simulated microgravity and spaceflight. (DOC 92 kb)

## Abbreviations

1G: Ground gravity
2-D: Two-dimensional
3-D: Three-dimensional
μG: Microgravity
AC: Agar culture or semi-solid media
ACT: Actinorhodin
CFU: Colony-forming units
DL: Diamagnetic levitation
EUEs: Experiment Unique Equipments
GBFs: Ground-based facilities
GS: Gramicidin S
HARVs: High-aspect rotating vessels
LSMMG: Low-shear modeled microgravity
MC: Microcystin
MRT: Minimum response time
NG: Normal gravity
PHB: Poly-β-hydroxybutyrate
RCCSs: Rotating cell culture systems
RED: Undecylprodigiosin
RPMs: Random positioning machines
RWBs: Rotating wall bioreactors
RWVs: Rotating wall vessels
SC: Suspension culture
SF: Spaceflight
SIMBOX: Science in microgravity box
SM: Simulated microgravity
SMG: Simulated microgravity
