# Supplementary material for: Effects of spaceflight and simulated microgravity on microbial growth and secondary metabolism
Source: Mil Med Res. 2018 May 14;5:18. doi: 10.1186/s40779-018-0162-9 (PMC5971428; doi:10.1186/s40779-018-0162-9)
Supplement: Supplementary file 1 — The different responses of microbial growth to simulated microgravity and spaceflight. (DOC 92 kb) [file 40779_2018_162_MOESM1_ESM.doc]

**Supplementary Material**

**Additional file 1:** **The different responses of microbial growth to simulated microgravity and spaceflight.**

**Table S1 Responses of microbial growth to simulated microgravity and spaceflight**

| Growth | Bacterial species and strains | Microgravity condition | Culture mode | Other condition |
| --- | --- | --- | --- | --- |
| Increased | *Escherichia coli* ATCC 4157^[7, 61, 64, 69, 70]^ | SF, SM | SC |  |
|  | *Escherichia coli* ATCC 26^[6]^ | SM | SC | Higher nutrient medium |
|  | *Escherichia coli* K12 MG1655^[56]^ | DL | SC |  |
|  | *Escherichia coli* ZK650^[65]^ | SM | SC |  |
|  | *Escherichia coli* K12 MC4100^[66]^ | SF | SC |  |
|  | *Escherichia coli* PhB405^[71]^ | SF | SC |  |
|  | *Escherichia coli* DH5α^[62]^ | SM | SC |  |
|  | *Bacillus subtilis* ATCC 6051^[7, 61, 67, 68]^ | SF | SC |  |
|  | *Salmonella typhimurium* BS-5 (P-22)/P-22^[72]^ | SF | SC |  |
|  | *Salmonella typhimurium* wild type χ3339, RpoS mutant χ4973^[18]^ | SM | SC | Minimal medium |
|  | *Pseudomonas aeruginosa* PA14, motility mutant strain^[8]^ | SF | SC | Nutrient-limiting |
|  | *Staphylococcus epidermidis* NCTC11047^[56]^ | DL | SC |  |
|  | *Ralstonia pickettii* (ISS isolate)^[78]^ | SM | SC | Non-motile (lacked flagella) |
|  | *Stenotrophomonas maltophilia* (ISS isolate 2)^[80]^ | SM | SC |  |
|  | *Acinetobacter radioresistens* (ISS isolate) ^[79]^ | SM | SC |  |
|  | *Sphingomonas paucimobilis* (ISS isolate) ^[79]^ | SM | SC |  |
|  | *Streptomyces coelicolor* A3(2) ^[95]^ | SF, SM | SC |  |
| Unchanged | *Escherichia coli* ATCC 4157^[7, 61, 67, 68]^ | SF, SM | AC |  |
|  | *Escherichia coli* ATCC 26^[6]^ | SM | SC | Minimal medium |
|  | *Escherichia coli* GC2852^[63, 71]^ | SF | SC |  |
|  | *Escherichia coli* ATCC 25922^[66]^ | SF | SC |  |
|  | *Escherichia coli* AMS6, AMS150, AMS171^[17]^ | SM | SC |  |
|  | *Bacillus subtilis* ATCC 6051^[7, 61, 67, 68]^ | SF, SM | AC |  |
|  | *Bacillus brevis* Nagano^[94]^ | SM | SC |  |
|  | *Salmonella typhimurium* Wild type χ3339, RpoS mutant χ4973^[18]^ | SM | SC | Complex medium |
|  | *Pseudomonas aeruginosa* PA14 wild type strain^[8]^ | SF | SC | Higher nutrient |
|  | *Pseudomonas aeruginosa* ATCC 29260^[74]^ | SM | SC |  |
|  | *Pseudomonas aeruginosa* UG2^[73]^ | SM | SC |  |
|  | *Staphylococcus aureus* RF1, RF6, RF11^[28]^ | SM | SC |  |
|  | *Streptococcus pneumoniae* TIGR4^[75]^ | SM | SC |  |
|  | *Saccharomyces cerevisiae* BY4743^[81]^ | SM | SC |  |
|  | *Saccharomyces cerevisiae* CMBSESA1^[13]^ | SF | AC |  |
|  | *Stenotrophomonas maltophilia* (ISS isolate 1)^[80]^ | SM | SC |  |
|  | *Pseudomonas ﬂuorescens* (ISS isolate)^[80]^ | SM | SC |  |
|  | *Sphingomonas paucimobilis* ATCC 10829^[79]^ | SM | SC |  |
|  | *Acinetobacter radioresistens* ATCC 49000^[79]^ | SM | SC |  |
|  | *Sphingobacterium thalpophilium* (ISS isolate)^[78]^ | SM | SC | Motile (had flagella) |
|  | *Streptomyces coelicolor* A3(2)^[95]^ | SF, SM | AC |  |
| Decreased | *Streptomyces clavuligerus* ATCC 27064^[77]^ | SM | SC |  |
|  | *Streptomyces* *hygroscopicus* ATCC 29253^[76]^ | SM | SC |  |
|  | *Streptomyces plicatus* WC56452^[27]^ | SF | SC |  |
|  | *Saccharomyces cerevisiae* Σ1278b^[13]^ | SF | AC |  |
|  | *Saccharomyces cerevisiae* Msn4 or Sfp1 deletion^[55]^ | DL | SC |  |

SF. Spaceflight; SM. Simulated microgravity; DL. Diamagnetic levitation; SC. Suspension culture; AC. Agar culture or semi-solid media
